# Depression and Severity Detection Based on Body Kinematic Features: Using Kinect Recorded Skeleton Data of Simple Action

**DOI:** 10.3389/fneur.2022.905917

**Published:** 2022-06-30

**Authors:** Yanhong Yu, Wentao Li, Yue Zhao, Jiayu Ye, Yunshao Zheng, Xinxin Liu, Qingxiang Wang

**Affiliations:** ^1^College of Traditional Chinese Medicine, Shandong University of Traditional Chinese Medicine, Jinan, China; ^2^School of Computer Science and Technology, Qilu University of Technology, Jinan, China; ^3^The First Affiliated Hospital of Shandong First Medical University & Shandong Provincial Qianfoshan Hospital, Jinan, China; ^4^Shandong Mental Health Center, Shandong University, Jinan, China

**Keywords:** human skeleton, kinect sensor, temporal convolution network, depression recognition, deep learning

## Abstract

Relative limb movement is an important feature in assessing depression. In this study, we looked into whether a skeleton-mimetic task using natural stimuli may help people recognize depression. We innovatively used Kinect V2 to collect participant data. Sequential skeletal data was directly extracted from the original Kinect-3D and tetrad coordinates of the participant's 25 body joints. Two constructed skeletal datasets of whole-body joints (including binary classification and multi classification) were input into the proposed model for depression recognition after data preparation. We improved the temporal convolution network (TCN), creating novel spatial attention dilated TCN (SATCN) network that included a hierarchy of temporal convolution groups with different dilated convolution scales to capture important skeletal features and a spatial attention block for final result prediction. The depression and non-depression groups can be classified automatically with a maximum accuracy of 75.8% in the binary classification task, and 64.3% accuracy in the multi classification dataset to recognize more fine-grained identification of depression severity, according to experimental results. Our experiments and methods based on Kinect V2 can not only identify and screen depression patients but also effectively observe the recovery level of depression patients during the recovery process. For example, in the change from severe depression to moderate or mild depression multi classification dataset.

## 1. Introduction

Depression is a widespread mental condition that affects about 260 million individuals globally (1). This is in contrast to the varying emotional and psychological stresses they face on a daily basis, which pose significant challenges to families and social burdens all over the world (2). Depression has become a major health problem, especially when it lasts for a long time and has a moderate or severe intensity. It can lead to severe maladaptation at work, school, and at home for the person who is affected. Depression can lead to suicide in the worst-case scenario. Current technology developments based on mobile internet interventions can give a variety of approaches for continuously monitoring individuals' psycho emotional status in high-risk situations. Automatic depression recognition attempts to provide valid indices of stress-related risk as part of inchoate evaluation or relapse prevention programs (3).

According to DSM-5 MDD (4), symptoms of clinical depression include markedly diminished interest or pleasure. It is accompanied by symptoms such as sleep disturbance, fatigue or almost loss of energy, loss of concentration, or daily indecision. These symptoms persist for more than 2 weeks. Human action has been discovered to represent patients' mental status, particularly the state of depressive disorders, as a natural, easily seen body activity (5). In terms of depression assessment, body expressions, gestures, and head movements may be as important visual norms for depression detection as facial expression recognition (6).

Depressive state were reflected in low energy, slow movement and expanded limbs and torso (7). Patients with major depressive disorder (MDD) increased muscle tension, which would affect their body movements. Besides, the observed during walking were reflected by reduced walking speed, reduced arm swing, reduced vertical head movements, abnormal hand movements, and head-down position in walking comparing to neutral, larger lateral swaying movements of the upper body and a more slumped posture and patients with depression showed larger reaction time variability (8, 9). Extrinsic stimulation tasks can improve patients' limb movements and gait, and predict the possibility of patients' recovery. Chang et al. (10) utilized Kinect combined with conditioning strategies to facilitate recovery therapy in patients with dyskinesia. Maggio et al. (11) found that exoskeleton gait training may help to achieve positive changes in BR, especially reducing psychological distress and perception of legs/thighs, demonstrating the beneficial role of exoskeletons in exercise rehabilitation for neurological disorders.

As an objective and easily accessible data source, although differences in patients with depression can be found through body signals, there are few methods to utilize them for depression identification. At the same time, there is no comprehensive uniformity of mental health status detection in stimulus tasks. Existing approaches can be divided into two categories: upper torso or relative limb portion movements (12). Relative body part skeletal movements represent orientation and displacement can be captured and extracted *via* Kinect (13) from the sensor's origin expressed in space coordinates. With the advantage of high performance, cost portability, and low cost, kinect may be a practical option to conveniently record body gestures in real time. High qualified and efficient computational models would be built which could recognize depression based on kinect-recorded skeleton data, rather than only find some motion gesture features relevant to depression. Using machine learning methods to automatically recognize the un-depression and depression, these original data driven features could not provide a high-level description of the gesture pattern of depression, such as turning or arm swing but may involve more potential information which would be calculated for recognition (14). In general, shaking or fidgeting behavior, psychomotor agitation or retardation, and diminished ability to concentrate have been considered signs of depression, the whole body, the upper body, or separate partial body involved in body gestures can contribute to the depression assessment. Our study has the following contributions: First, we innovatively proposed a natural stimulus skeleton imitation task and used Kinect V2 to complete the data collection work. Second, we proposed the TCN-ResNet18 model for binary classification (depressed and non-depressed) and depression severity identification (four levels: severe, moderate, probable, and non-depressed), and got excellent results.

## 2. Related Studies

It is well-known that depression symptoms through a variety of verbal and non-verbal signs. Researchers have focused their attention on a visual and linguistic indications for assessing depression. This part introduces related work on facial features, audio features, body features, and Kinect-based rehabilitation training.

### 2.1. Faces Feature

As a standard criterion for depression recognition, micro-facial expressions aim to make timely depression detection and usually need elaborate specialist training. Extracting micro-expressions in different methods from the face attempt to support depression decisions. Given that depression is associated with emotional expression and greater persistence variability reducing a neutral expression, it is a significant feature that the whole face is concerned as basic emotional expressions displaying that will be extracted. Creating more datasets with a broader participant group and stimuli task is required to ensure the subconscious micro-expression detection for depression recognition can be expanded. Davison reported a dataset SAMM that had performed as a good resolution, which included a newly developed diverse participants spontaneous micro-facial movement dataset and used the Facial Action Coding System (15). Wang introduced an effective method for automatic classification of patients with depression, which used the key facial features extracted from human specific active appearance characteristics based on visual information where the video was collected from the control group of patients with depression (16). With the deep learning development, some studies achieved competitive consequences using visual data for depression detection (17, 18).

### 2.2. Audio Feature

Despite the rising interest in Automatic Depression Detection (ADD), existing reviews were varying in their specific perspective, and some attempts were in further assessment of methods and results. Depressed people behave differently from normal people which can be detected in the audio of the patient. Studies have shown that depressed people tend to speak anxiously in short phrases and monotonously, engage less in verbal communication and few eye contact. For instance, using audio data for depression detection by interpreting the responses of individuals to a variety of questions, an effective automatic detection method must understand cues of queries from varying prognostic values like professionals. Some audio-based depression detection studies got admirable progress (19–21), and several studies detailed that multi-modality solutions confused with vocal and visual data showed higher performance than those of either single modality (22–24).

### 2.3. Body Feature

Body signs, in general, have been shown to convey manifestations of depression, existing studies can be classified as relying on either upper body or relative body part movements. Approaches proved upper body sportive traits or relative body part movements, could be extracted as features for depression classification (6, 25). As a natural, it is easy to be observed from body movements that human gait has been found to reflect the walker's mental aspects of depression. Zhao et al. (26) found that natural gait can effectively measure anxiety and depression. Fang et al. (27) found significant differences in several spatiotemporal, kinematic, and postural gait parameters such as walking speed, stride length, head movement, vertical head posture, arm swing, and body sway, between the depressed and non-depressed groups. Although there has been much evidence that depression could be reflected by human kinematics characteristics, which could be a clear objective, easily accessible data source, the methods of skeletal-based mental health state detection have not yet been fully established. Depression can be identified by various non-verbal signs (28). Stimulus changes in the tonic activity of muscles, as well as posture challenges, and kinematic parameters computed, often mirror the characterized depression (29). In action observation, it was discovered that neutral and depressed participants had different hand motions and head-down attitudes, with depressed patients having decreased velocity, reduced amplitude, and greater swing time variability (30). Microsoft Kinect for depth and skeleton data recording has been widely and successfully used in human action recognition (31). Kinect is used as a powerful tool for tracking and capturing people's footsteps.

### 2.4. Multimodal Feature

Multimodal depression recognition is a hot issue in current research. Different modes have different ways of expression and different angles of looking at things, so it will produce certain information complementarity. Ye et al. (32) proposed a multimodal fusion method based on the depth spectrum feature and word vector feature, which uses deep learning to detect depression and obtains excellent results. Ray et al. (33) proposed a new multimodal depression prediction network based on multi-level attention, which is used to strengthen the training of the model by selecting the features with the greatest attention. Yin et al. (34) proposed a multimodal method and hierarchical recursive neural structure and introduced an adaptive sample weighting mechanism to predict the severity of depression.

### 2.5. Kinect-Based Rehabilitation Training

A large number of studies have proved that the use of Kinect can effectively help patients with limb motor function rehabilitation. Bao et al. (35) used Kinect to provide rehabilitation training for stroke patients with lower extremity dysfunction. Fugl-Meyer assessment and Wolf motor function test scores of hemiplegic upper limbs in patients with stroke were significantly increased at 3 weeks after training and at 12 weeks of follow-up. Almasi et al. (36) evaluated the application of Kinect-based virtual rehabilitation for upper extremity motor recovery in patients with chronic stroke and demonstrated that range of motion was also improved in shoulder flexion and shoulder horizontal adduction. Aşkın et al. (37) found that VR training with Kinect helped improve UE motor function and AROM in patients with chronic stroke. It is worth noting that Song and Park (38) found that Kinect's virtual game can effectively alleviate the psychological problems of patients with stroke, and found that the patients' depression levels improved through the BDI scale. Therefore, this article proposes a Kinect-based limb stimulation task to provide a new measure for the rehabilitation of patients with depression.

Using Kinect for skeletal data recording to establish a depression detect method, strong computational models are necessary that could detect depression efficiently, rather than only find some relative features (39). However, currently, body-based utilities for depression assessment have only been found in fewer research-related projects and evaluated their feasibility for applications in the general population. Compared to traditional methods, significant progress has been achieved by deep learning, but two key points were still worth digging into the execution, a basic temporal feature distinguishing and long-term dependency modeling, limiting the further increase in performance of skeleton-based depression prediction. A novel deep learning architecture, temporal convolution network (TCN), is proposed for human action recognition (17, 40, 41).

Many subjects of common interest were discussed in the present review, but within the broader field of pattern recognition and skeletal data analysis, and barely specifically focusing on depression assessment. Because this article is on body language signals, we briefly reviewed the recent relevant methods in the body capturing channel according to the feature extraction and preprocessing, stimulus tasks design, handcrafted dataset based, and deep learning detection.

## 3. Data Collection

### 3.1. Site and Apparatus

**Figure 1:**
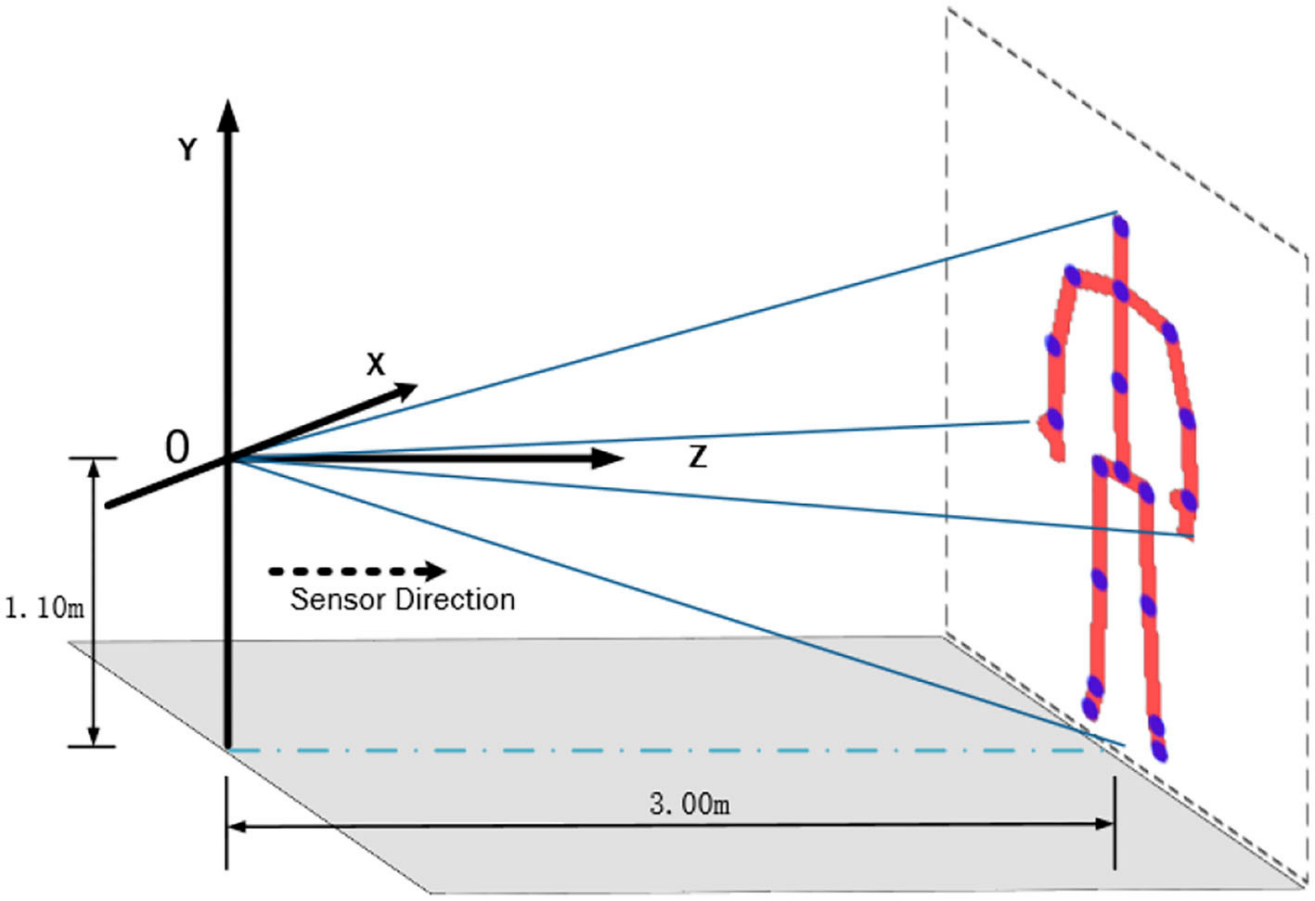
Illustration of an experiment site. According to the basic parameters of Kinect, it is the best layout to fully catch the participants that Kinect was installed on a bracket 1.1 m above the ground, which was standing 3 m in front of the equipment and facing the Kinect.

The data collection was completed in the special lab of the Shandong Mental Health Center. Participants were in a room that was setup to keep interaction with researchers to perform the stimulus task and allowed them to become comfortable in the experimental surroundings. Participants executed the stimuli task following audio, which was covered by researchers. In this experiment, we used an i7-6700 CPU with a Win-10 OS computer for recording and Kinect device control. Kinect took advantage of its low price and depth sensing capability, which were used to capture the participants' skeletal data. The skeleton joint coordinate streams during recording were generated by Kinect Studio V2 software with a frame rate of 30Hz. According to the basic parameters of Kinect, participants were standing 3 m in front of the Kinect to complete the collection procedure in order to capture the whole body movement. To avoid the influence of illumination and improve the recognition rate of the equipment, we set a green curtain as human skeleton detection background. The green curtain is helpful to reduce the interference of other factors and make the model extract more effective features, but the effect of the model may decline in other backgrounds. Kinect is a very easy device to handle and equip, as the data collection experiment layout shown in Figure 1. Before the experiment started, clinicians would explain the rules to the participants until the participants understood the procedure considering some patients' conditions. Then the participants entered the located site in the laboratory to adapt to the environment in advance.

### 3.2. Participants and HAM-D Assessment

The most difficult aspect of this study is finding participants, especially patients with depression. All the patients with depression will be evaluated by the professional psychiatrists, and they will make pre-screened according to the patients' treatment condition. In this study, we gathered 85 patients with depression from the Shandong Mental Health center and 125 normal people as a control group from society recruited totally and their ages were between 18 and 65. There were 210 participants in this experiment, which everyone would be accessed by psychiatrists referred to the HAM-D assessment standard (42). Hamilton Rating Scale for Depression (HRSD) (43), also called the Hamilton Depression Rating Scale (HDRS), abbreviated HAM-D, is used to provide an indication of depression and a guideline for recovery evaluation, which includes a multiple item questionnaire. By probing mood, suicide ideation, insomnia, anxiety, agitation or retardation, feelings of guilt, weight loss, and somatic symptoms, the questionnaire is used to rate the severity of their depression, which is designed for adults' assessment. Hamilton Depression Rating Scale has been criticized for use in clinical practice as it places more emphasis on insomnia than on feelings of hopelessness, self-destructive thoughts, suicidal cognition, and actions. In this experiment, we used the version containing 24 items (HDRS-24), and the total score is compared to the corresponding descriptor. Participants were interviewed to assess symptom severity according to the scales: Not depressed (0–8), Probable (8–20), Depressed (20–35), and Very severe (>35). The participants were assessed according to diagnostic criteria for the WHO (ICD-10) by years of experienced psychiatric clinicians. Each participant's assessment time was about 15 min. The experiment protocol had obtained permission from the Shandong Mental Health center and participants.

**Figure 2:**
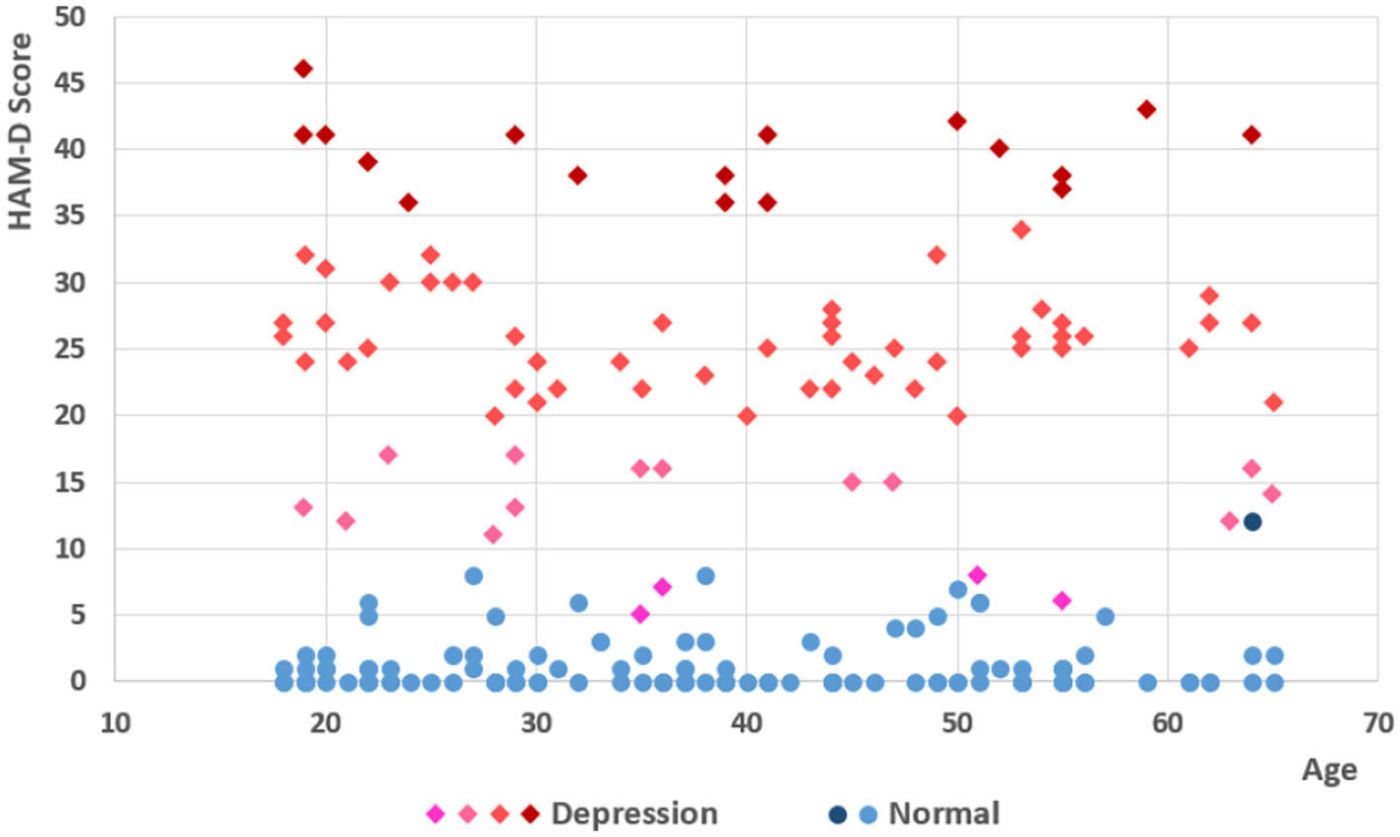
Score and age of participants. There are 85 patients with depression and 125 normal participants in the experiment. Following the scales of HDRS-24, participants were divided into four different depressed levels, not depressed (0–8), probable (8–20), mid depressed (20–35), and very severe (>35), which includes 16 severe, 50 medium, and 14 probable (13 patients and 1 normal participant) and 130 non-depression (4 patients and 126 normal participants).

Aging seems to be a critical reason for the human capacity for human action ability limitation. We set the baseline age of participants from 18 to 65, which the depression group's average age is 39.7 (var = 211.4, std = 14.5) and the normal group was 38.5 (var = 190.7, std = 13.8), as shown in Figure 2. The good sensitivity and specificity of HAM-D for detecting depression had been proved by many previous studies, with the usual cut point > 8 for scales. Some of the patients get scores <8, as the psychiatrist analyses that they recovered by hospitalization in the ward, while they had been treated for nearly 2 weeks in particular.

### 3.3. Stimulus Task Design

**Figure 3:**
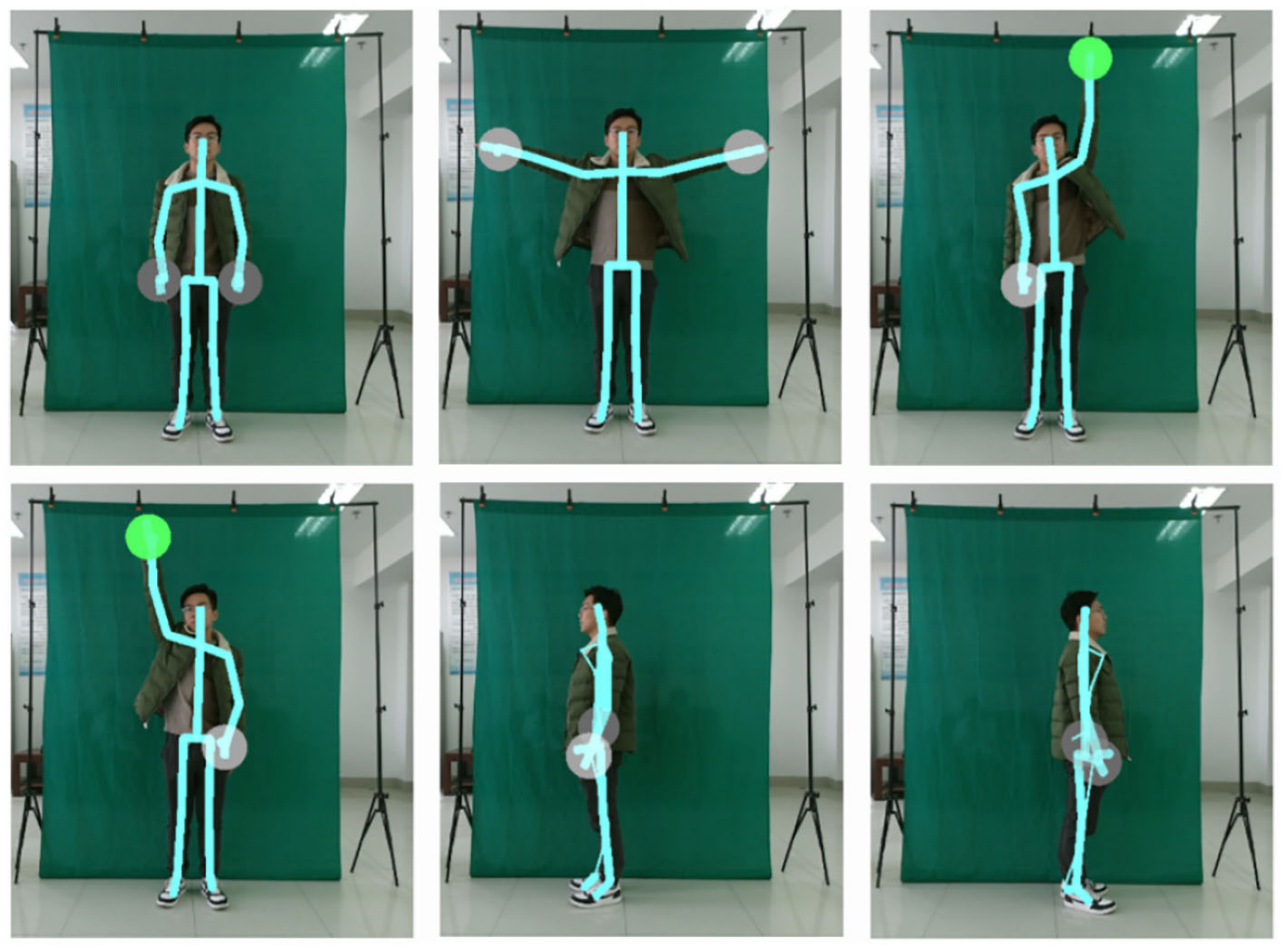
The keyframe of stimulus task action. Considering the patient's physical condition, to eliminate irrelevant factors like education, profession, the stimulus task we designed is very easy to execute, which contains lift two-hands, lift left hands, lift right hands, turn right, turn left.

To eliminate irrelevant factors like education and profession, we designed a series of directives as simple as possible. Using the Kinect-default 3D coordinates with the sensor position as the initialization may cause non-negligible deviation in the stimulating progress, due to the different positions relative to the Kinect camera of different participants during recording participants' responses. All of the participants would follow the action direction, standing at the specified location. The stimulus task was separated into five parts, in which all the participants were asked to lift two hands, lift left hands, lift right hands, turn right, turn left and reset without intentional previous training, as shown in Figure 3. Kinect sensors record continuously for 60 s to ensure sufficient high-quality skeletal motion capture.

Since the purpose of this experiment is to identify patients with depression and observe the level of recovery in patients with depression, we will complete the data collection of the Stimulus Task in two stages. The first is the initial stage, we will collect data on the subjects of the case group and the control group on the basis of the Stimulus Task. Second, in the following 4 weeks and 12 weeks, data collection was performed again, and this part of the study is currently in progress.

### 3.4. Skeleton Data Extraction

The skeleton data recordings of participants' activity from Kinect were the 3-dimensional accelerations of the 25 body key joints. The human torso is the most reliably detected area, even under heavy occlusions, as it can be accurately estimated based on other features' 3D positions. Skeletal sequences are mapped to the motion capture coordinate space (*X*, *Y*, and *Z*-axis) and tetrad (*Rx*, *Ry*, *Rz*, and *Rw*). It is worth noting that 3D refers to the spatial position of each joint of the target detected by Kinect (*x*_*t*_, *y*_*t*_, *z*_*t*_). For all the relevant human skeleton joint points to be locked in the experimental target object in any stimulus task time t, there are:


(1) $$\left(x_{t},y_{t},z_{t}\right)=\{\begin{array}{l} x_{\text{right }}\leq x_{t}\leq x_{1\text{eft }}\\ y_{\text{low }}\leq y_{t}\leq y_{\text{top }}\\ z_{\text{front }}\leq z_{t}\leq z_{\text{back }} \end{array}$$

The spatial coordinate position of each human skeleton node (*x*_*t*_, *y*_*t*_, *z*_*t*_). In time t, the obtained quaternion is calculated automatically by Kinect [(Rx_t_, Ry_t_, Rzt_t_), Rwt_t_].


(2) $$\{\begin{array}{l} \text{x}=\sin(\text{y}/2)\cdot \sin(\text{z}/2)\cdot \cos(\text{x}/2)+\cos(\text{y}/2)\cdot \cos(\text{z}/2)\cdot \sin(\text{x}/2)\\ \text{y}=\sin(\text{y}/2)\cdot \cos(\text{z}/2)\cdot \cos(\text{x}/2)+\cos(\text{y}/2)\cdot \sin(\text{z}/2)\cdot \sin(\text{x}/2)\\ \text{z}=\cos(\text{y}/2)\cdot \sin(\text{z}/2)\cdot \cos(\text{x}/2)-\sin(\text{y}/2)\cdot \cos(\text{z}/2)\cdot \sin(\text{x}/2)\\ \text{w}=\cos(\text{y}/2)\cdot \cos(\text{z}/2)\cdot \cos(\text{x}/2)-\sin(\text{y}/2)\cdot \sin(\text{z}/2)\cdot \sin(\text{x}/2) \end{array}$$

**Figure 4:**
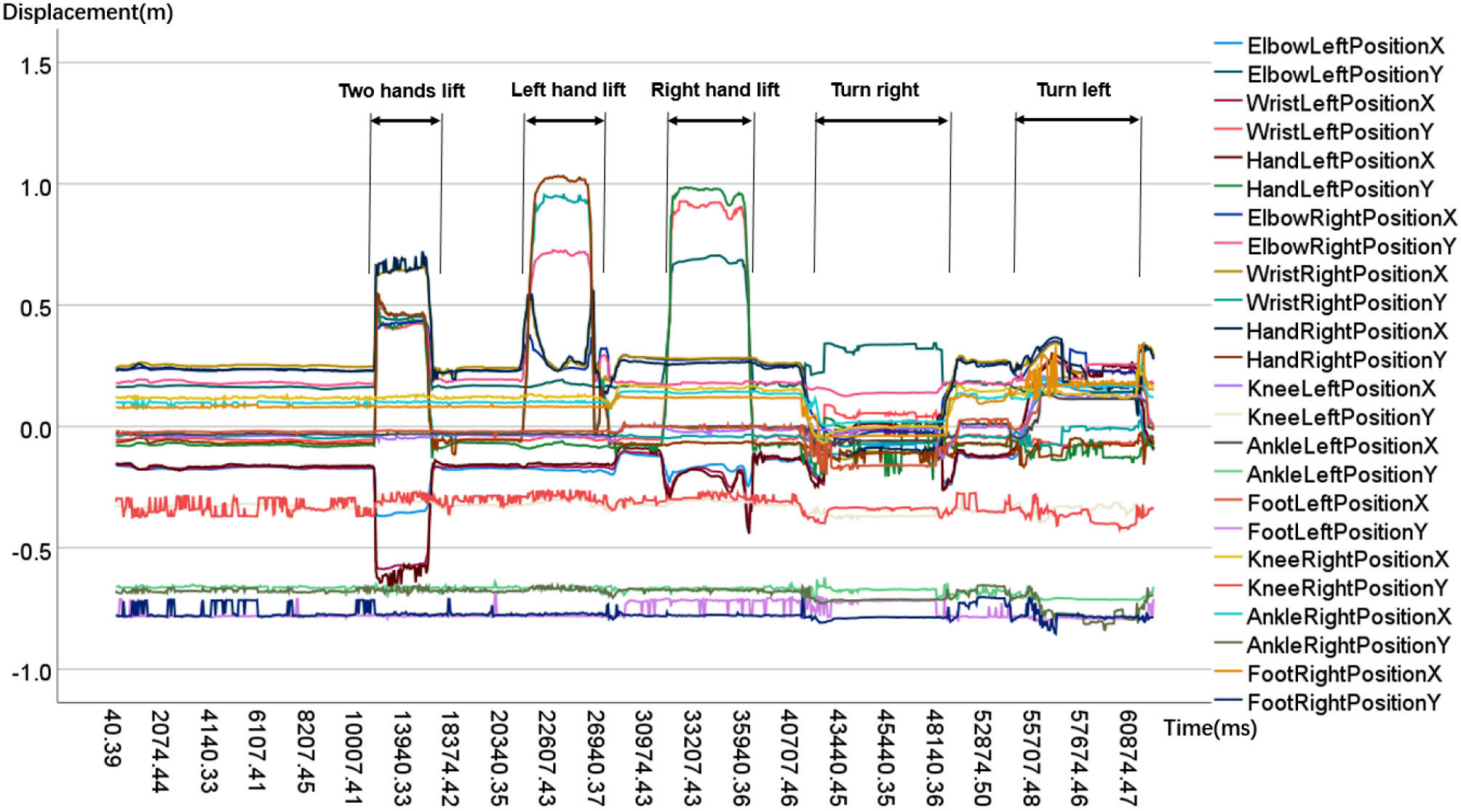
Time sequences of partial body joints. From the sample's key joints kinematic features, Upper body joints' movement range like hand, elbow is apparently bigger than lower body joints' like foot, ankle.

Using the rigid transformation obtained from the calibration. each body event index containing 25 joints will be recorded by the coordinate system with the position. Thus, joints skeleton data in the time sequences follows:


(3) $$\begin{array}{r} d_{jt}=\left[x_{jt},y_{jt},z_{jt},Rx_{jt},Ry_{jt},Rz_{jt},Rw_{jt}\right] \end{array}$$

where *j* is the joint index, and *t* is the timing when the body index was captured. Particularly, some joints do not exist in tetrad coordinate, such as the head and foot joint in the original Kinect recording skeletal data. Skeleton extracting platform was available in many development environments. When we analyzed the joints' kinematic features, we found that only a few participants deviated slightly from the stimulus motions they were supposed to follow. We did not include these data in our data set since they were excluded. We draw a sample's partial joints in terms of the X and Y coordinate space axis, shown in Figure 4.

In order to extract and pre-process skeleton data custom, we developed a skeleton-extractor program based on .net core 3.0. As a non-intrusive extracting method, shortening the necessary recording sequence would improve its practical value, so we tried to select crucial series from each participant during sampling. We divided the 1 min recording file of each Kinect into stable segments, based on whether the direction of audio playing was done.

### 3.5. Data Preprocessing

Before feature mapping, we noticed that the skeletal data are flexible and variant in the sequence, which causes great difficulties in joints relationship and decisive kinematic information analysis. In more complicated cases, normalization referred to more sophisticated adjustments where the intention is to bring the entire probability distributions of adjusted values into alignment. In the case of normalization of scores in depression assessment, there may be an intention to align distributions to a normal distribution. A different approach to normalization of probability distributions is quantile normalization, where the quantiles of the different measures were brought into data standardize. Feature scaling is used to bring all values into the range [0,1]. We use the data normalization method following the below equation:


(4) $$\begin{array}{r} {\text{X}}_{\text{i}}^{*}=\frac{{\text{X}}_{\text{i}}-{\text{X}}_{\min}}{{\text{X}}_{\max}-{\text{X}}_{\min}} \end{array}$$

### 3.6. Dataset Handcrafted

We handcrafted the preprocessed skeletal dataset of the entire body. The datasets would be implemented for the skeleton depression recognition, which included binary classification and multi classification.

In the binary classification dataset, according to the HDRS-24 assessment standard and the psychiatrist's advice, we followed the rules to kick off some samples: depression patients' group HDRS-24 score <8 and normal group HDRS-24 score >8. All the participants accepted the HAM-D assessment, while 81 valid depression samples and 124 valid normal samples with HAM-D scores were satisfied, where the depression group got a higher HAM-D score (mean = 26.8), and the normal group got a lower score (mean = 1.1), as shown in Table 1. Following the psychiatrist's evaluation and suggestion, 4 patients and 1 normal original data were removed. There are 81 depression and 124 non-depression samples in the final binary classification dataset, then the sorted depression and normal group's age and HAM-D score are similar to unscreened, as is shown in Table 1.

**Table 1:** Age and HAM-D score in the binary groups.

**Participants groups**		**Original (210)**		**Screened (205)**	
		**Mean**	**Std**	**Mean**	**Std**
Depression	Age	39.4	14.7	39.0	14.3
	Score	26.6	13.6	26.8	8.7
Normal	Age	38.5	13.8	38.3	13.6
	Score	1.4	2.1	1.1	1.8

Participants were categorized into four groups in the multi classification dataset, which included all samples: non-depression, probable, medium depression, and severe. Participants were divided into four groups, following the scales as not depressed (0-8), probable (8-20), mid depressed (20-35), and very severe (>35). The whole dataset contains 210 samples, which includes 16 severe, 50 medium, 14 probable, and 130 non-depression samples, as is shown in Table 2.

**Table 2:** Age and HAM-D score in the multi-classes groups.

**Groups**		**None (130)**	**Probable (14)**	**Medium (50)**	**Severe (16)**
Score	Mean	1.2	14.0	25.7	39.6
	Std	2.0	2.0	3.5	2.7
Age	Mean	38.5	40.6	39.7	37.9
	Std	13.6	17.3	13.9	15.6

## 4. Approach

### 4.1. Dilated Temporal Convolution Network

**Figure 5:**
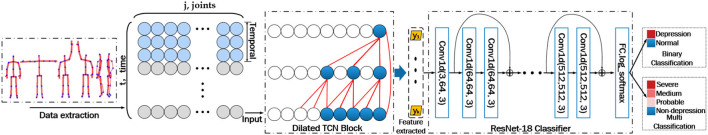
Depression recognition model TCN-ResNet18. The body skeleton for each frame. The *d*_*jt*_-dimensional skeleton vector for time index, constructed by concatenating the 3D and tetrad coordinates of each body joint. The model contains three temporal blocks and one designed residual-18 layers network. The model will be applied to binary and multi depression prediction. In order to match the output dimension of the TCN block, we rebuilt the ResNet18 in single-dimensional convolution architecture in the ResNet-18 Classifier.

As demonstrated in Figure 5, our method allows us to learn kinematic skeletal sequence features for a depression diagnosis.

Time convolution network (TCN) is a sequence recognition model. It extracts local spatiotemporal features from sequence data and performs classification. TCN was an effective and trainable temporal encoding network, which generated long-term representation from sequential skeletal data. The input to the model will be a set of body skeleton event time indexes, such as those output from a residual dilated convolution network, for each frame of Skeletal features. Note that the number of time steps *T* may vary for each skeleton sequence. The sequence modeling task is defined as follows. Given an input temporal skeletal sequence *x*_*t*_ of length *t*, where *x*_*t*_ is an observation for the time step. The prediction at each time step is defined as *y*_*t*_, where it should depend only on past observations and not future observations. For skeletal characteristic leaning, we need designed filters of standard convolution to fix temporal understanding capable of modeling suitable temporal windows ideally modeling the entire skeleton sequence.

To learn the significant skeletal characteristic, following the study of (40, 44), We employ dilated convolutions that enable an exponentially large temporal context scale at different layers of the network. For input body event time indexes *x*_*t*_ of length *t* and a filter *F*:{0, 1, …, *k*−1} → *R* for some filter size *k*, the dilated convolution operation *G* on element *s* of the skeletal sequence in each temporal layer is:


(5) $$\begin{array}{r} F\left(s\right)=\left(x{⊗}_{d}f\right)\left(s\right)=\overset{k-1}{\underset{i=0}{\sum }}f\left(i\right)\cdot x_{<\left(T-di\right)} \end{array}$$

where *d* is the dilated convolution scale and ⊗ means the operator of the convolution. With different dilation scales *d* and kernel size *k*, the network allows us to model the whole sequence. When *d* = 1, a dilated convolution reduces to a standard convolution. If the dilated convolution fits with *d* = 2*i, then the input layer starting receptive field will be covered by a filter at time index *t*, layer *i* can be described as follows:


(6) $$\begin{array}{r} s=\max\left(0,t-\left(2^{i+1}-1\right)*\left(l-1\right)\right) \end{array}$$

where *s* is the start time index step of the receptive field at the input layer and *l* is the size of the kernel filter. The final output *Y* of each temporal residual block is summed using a set of end-to-end skip connections. Let $X={\left[x_{1},x_{2},\ldots ,x_{T}\right]}^{⊤}$ be the input feature vector of length time index step *t* for 1 ≤ *t* ≤ *T*. Suppose each time index step will be presented as a prediction *y*_*t*_, then the steps precision result is:


(7) $$\begin{array}{r} Y={\left[y_{1},y_{2},\ldots ,y_{T}\right]}^{⊤} \end{array}$$

Where the activation function is set as Relu in all blocks.

### 4.2. TCN-ResNet18 Architecture

We proposed an end-to-end TCN-ResNet18 for skeleton-based depression recognition. The overall network structure is shown in Figure 5. The sequential skeleton features are stacked temporally across the entire stimulus task sequence to form the input block, which is later imported into the ResNet18. Specifically, our network employed ResNet18 to improve the classification capacity. The output *Y* of the final temporal residual block will be changed the dimension into 768 dimensions through a linear layer we set and reshaped as [batch size, 3, 16, 16]. It is then fed into the ResNet18 block. (45). An important design principle of the residual network is that when the size of the feature map is reduced by half, the number of feature maps is doubled, which keeps the complexity of our network. The final output *C* of ResNet18 is summed following the residual computation:


(8) $$\begin{array}{r} C=\text{LogSoftmax}\left(Y+F\left(Y\right)\right) \end{array}$$

where *C* is the precision result. We employ our model for binary-class and multi-class depression recognition experiments. The final prediction label for each object is given by vector *C*_*p*_∈{0, 1}, where the result will be predicted in the binary classes: depression or non-depression, such as the true class Depression is 1 and false class Non-depression is 0. Following the original ResNet18 architecture, we rebuilt our network with single-dimensional convolution computation to match the TCN block output dimension.

Considering the high-intra variance nature of joint motion, we notice that not all body motion sequences and kinematic features extracted by TCN contain the most discriminative information. Irrelevant motion sequences and features may bring in unnecessary noises. Given this description, along with the final TCN output, we introduce the ResNet18 to make a decision on the generated features. The classifier block can track the origin of relevant information by compressed sensing once the internal layer and fully connection layer estimates are available. Because of the forward dilated temporal convolutional residual blocks feeding, the architecture of the model can well identify the relevant body event index series segment and the pivotal body joint kinesiology features. Given a set of skeleton sequence data, the precision result can be calculated by the efficient model we proposed.

### 4.3. Implementation Details

During the training phase, the batch size was set to 32 at the end of each training dataset to keep the same sequence length within each batch. We used Adam for optimization by minimizing the loss. There are 3 temporal blocks and one 18-layers residual block in our network, and we set the dropout rate to 0.2. We initialized the learning rate of 0.0025 for compatible parameters to achieve the best performance. While the exact skeletal sequences with the proposed network, we employed an Nvidia GTX-1070 for 100 epochs. Our implementation was executed on PyTorch.

## 5. Results

We have evaluated our proposal on two handcrafted datasets, a binary classification dataset and multi classification dataset. Experimental results show that TCN-ResNet18 is the state-of-the-art method for depression detection.

### 5.1. Binary Classification

Detecting the diagnosed-depressed individuals from the non-depressed ones was a representative binary classification problem. Comparison with other classical machine learning and deep learning methods in the skeletal modality. In order to evaluate the classification performance of extracted skeletal features, this study employed classical machine learning algorithms, i.e., Support Vector Machine (SVM) and Random Forest (RF) some of which have been successfully applied to depression analysis. Since recurrent neural networks (RNN) were the most used sequence data in the previous study, we set the deep learning algorithms, like long short-term memory neural network (LSTM) for comparison, and we also applied the classic convolution neural network like VGG-13 (46).

Experiments were conducted on the handcrafted skeleton dataset, which was separated 70% for training and 30% for testing. The precision, sensitivity, and specificity were calculated as the measurement of the predictive accuracy, which is commonly used to evaluate classification methodologies. Precision is the fraction of the cases with depressive symptoms among all individuals detected by the model; sensitivity refers to the test accuracy of scored-depressed groups, and specificity refers to the test accuracy of the non-depressed group. We compute the precision of sensitivity and specificity for true positives, true negatives summed over all classes. We displayed performance comparisons of our proposed network with mentioned methods in Table 3.

**Table 3:** Comparison between TCN-ResNet18 and other methods, where Ours refers to TCN-ResNet18.

**Methods**	**Accuracy (%)**	**Sensitivity (%)**	**Specificity (%)**
SVM	58.1	41.7	68.4
RF	64.5	50.0	73.7
VGG-13	59.7	45.8	68.4
LSTM	66.1	54.2	73.7
TCN	71.0	58.3	78.9
Ours	75.8	66.7	81.6

Although the effectiveness of the third-party assessment as a judgment tool in accessing depression severity has been well-proved in studies, the procedure itself cannot be used as the main diagnosis method. With the cutpoint-8 for the classification scales, few patients achieved the level of non-depression, which means that there may be a small quantity of “false” patients with depression disorders in the experiment. The current study used interview assessment based scales of depressed classification criteria but not a clinical diagnosis of either. In the skeleton based depression recognition procedure, we found abnormalities in several kinematics characteristics including postural, amplitude, and spatial-temporal parameters. Although body kinematic signs have been shown to convey manifestations of depression in general, our approach is able to differentiate between a depressed and non-depressed object with relatively high accuracy. The recognition accuracy rate of our proposed model achieved 75.8%. The experimental results showed a conspicuous enhancement of recognition rates compared with the listed methods in Table 3. Compared with the best performance method TCN of the base line, we could observe that our model brings a 4.8% accuracy improvement on the test dataset.

**Figure 6:**
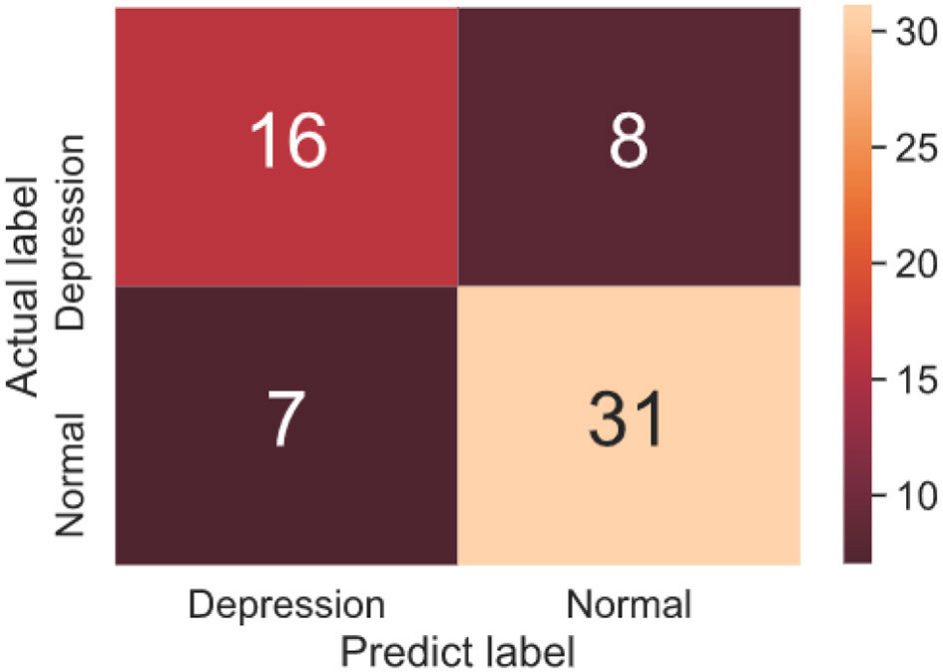
Confusion matrix of binary classification testing set.

The confusion matrices of the testing dataset are shown in Figure 6. It could be observed from the confusion matrix that the depression and non-depression were usually misclassified. From Table 3, the proposed model achieved a 66.7% sensitivity rate and 81.6% specificity rate on the test dataset. Because the case and control groups did not have the same number of participants in the experimental setting. As a result, there is a distinction between specificity and sensitivity. In our study, specificity is generally higher than sensitivity.

### 5.2. Multi Classification

**Figure 7:**
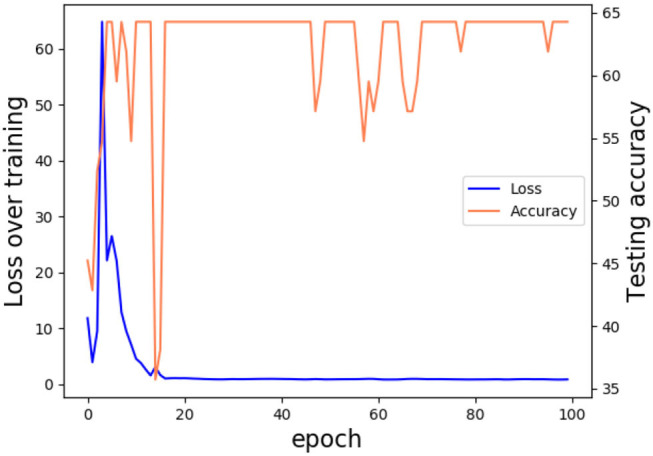
Loss and testing accuracy curve. Trained in 100 epochs, our model got 64.29% accuracy and the loss rate stabilized at around 0.7.

Referring to the HDRS-24 assessment principle, we employed our TCN-ResNet18 in the multi classification depression detecting work, which recognized different depression severities of participants. According to the scale HAM-D score statistics and standards, participants were divided into four groups non-depression, probable, medium depression, and severe. Experiments were conducted on the multi-classes dataset, which was separated 80% for training and 20% for testing. Our model has also achieved good results in multi classification tasks, which gets 64.29% accuracy. As expected, the TCN-ResNet18 performs well in the multi depression classification task. This verifies that using relevant skeleton information is important for best performance achieving. The loss and testing accuracy curve suggest that the confidences for the model are reliable, as is shown in Figure 7.

**Figure 8:**
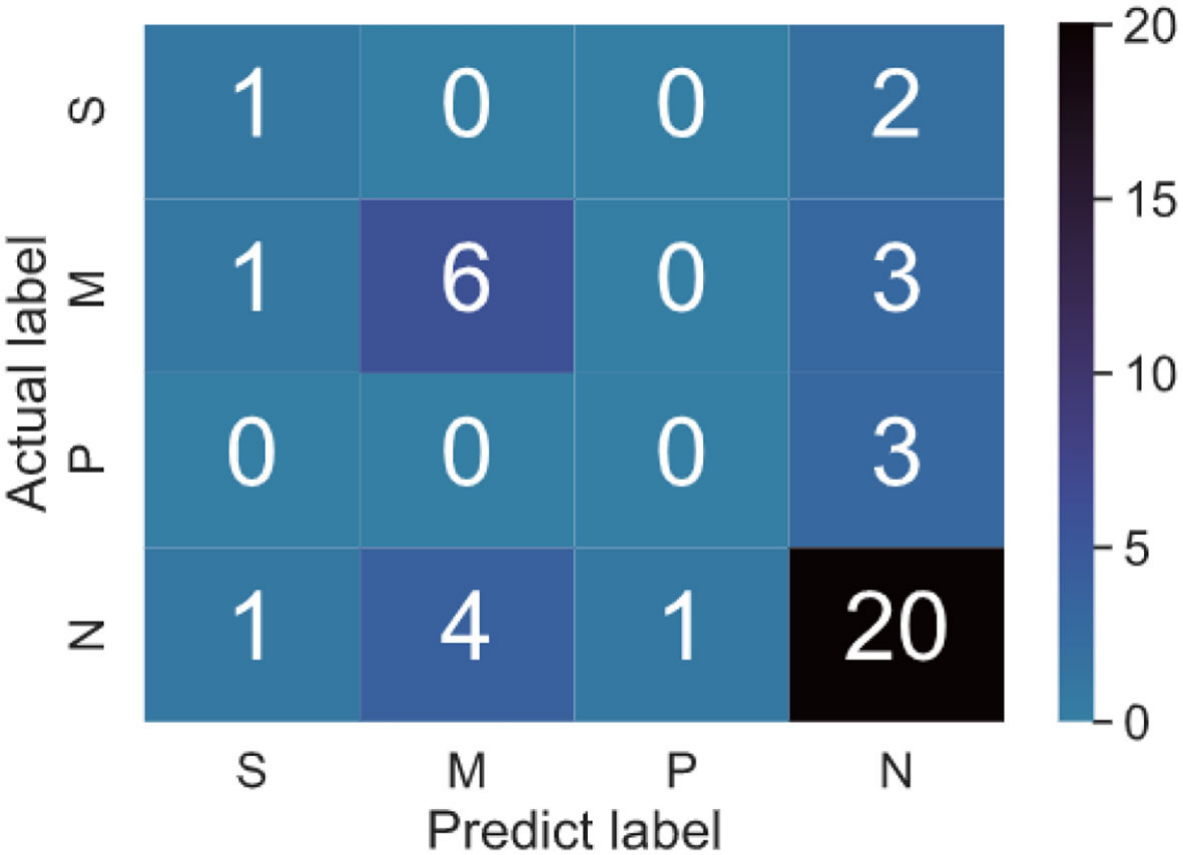
Confusion matrix of binary classification testing set. There are four groups in the multi classification dataset. Four classes contain S-severe, M-Medium, P-probable, and N-none depression. For example, 4 in N rows and M columns in the figure indicates that the real label is none depression, and the predicted label is Medium depression.

The apparent differences in severity of depression among the model effectiveness in detecting bring us more information about the accuracy of the classifier. The multi classification confusion matrices of the testing dataset are shown in Figure 8. Medium depression and non-depression are normally classified, because of the uneven distribution of samples. In the depression severity prediction task, medium depression and non-depression are normally classified, because of the uneven distribution of samples. Despite those limitations of the result, it suggests the potential for skeletal depression detection. An individual's skeletal kinematic information is objective and could be obtained repeatedly, while the participant's finishing HAM-D assessment is a subjective procedure.

The effective precision model was built through the deep learning method, based on the sequential features directly extracted from the original Kinect-3D coordinates of the participant's 25 body joints. The presented skeletal feature descriptors in this research appeared to be based on specific motion direction, and slightly subjective evaluations (HAM-D), which restricted the different features integrating capacity into the employed predictive model. The kinematic features in our study may not provide enough evidence of participant's reflection, however, it could cover the information of an individual's psychological status reflected during the stimulus task more comprehensively. Our results showed the validity of the computing model based on the low-level features in recognizing questionnaire measured severities of depression and showed the potential of this data-driven approach in the field of psychometrics.

## 6. Conclusion

Using the Kinect V2 to record participants' kinematic skeleton data, we imported the data into our model which contained the original Kinect-3D and tetrad coordinates of the participant's 25 body joints. We improved the TCN network, TCN-ResNet18 a novel skeleton temporal convolution network combined for depression prediction based on skeletal data, which built a hierarchy of temporal convolution groups with different dilated convolution rates and residual blocks across temporal scales to capture important crucial skeletal features. Moreover, the ResNet18 network is further applied to enhance the discriminative power of full scale representation. The effectiveness of the proposed method is demonstrated by the state-of-the-art performance achieved on the depression benchmark in the visual modality. In addition, this model shows good generalization executed on the binary and multi classification tasks. In the follow-up study, we will continue to complete the 4-week and 12-week follow-up data collection of the subjects, and improve the experimental method and the overall model performance.

## Data Availability Statement

The datasets presented in this article are not readily available because of ethical and privacy restrictions. Requests to access the datasets should be directed to the corresponding author/s.

## Ethics Statement

The studies involving human participants were reviewed and approved by Ethics Review Committee of Chinese Registered Clinical Trials. The patients/participants provided their written informed consent to participate in this study. Written informed consent was obtained from the individual(s) for the publication of any potentially identifiable images or data included in this article.

## Author Contributions

YY: experimental design, data collection, and manuscript writing. WL, JY, and QW: data collection, data processing, programming, and manuscript writing. YZha: data collection and data processing. All authors contributed to the article and approved the submitted version.

## Funding

This study was supported by the Shandong Provincial Natural Science Foundation, China (Grant Nos: ZR2021MF079 and ZR2020MF039). The National Natural Science Foundation of China (Grant No: 81573829). The Key Research and Development Program of Shandong Province (Grant No: 2020CXGC010901).

## Conflict of Interest

The authors declare that the research was conducted in the absence of any commercial or financial relationships that could be construed as a potential conflict of interest.

## Publisher's Note

All claims expressed in this article are solely those of the authors and do not necessarily represent those of their affiliated organizations, or those of the publisher, the editors and the reviewers. Any product that may be evaluated in this article, or claim that may be made by its manufacturer, is not guaranteed or endorsed by the publisher.
